# The Endemic Amid the Pandemic: Seeking Help for Violence Against Women in the Initial Phases of COVID-19

**DOI:** 10.1177/0886260521997946

**Published:** 2021-03-10

**Authors:** Susan B. Sorenson, Laura Sinko, Richard A. Berk

**Affiliations:** 1 University of Pennsylvania, Philadelphia, USA

**Keywords:** COVID-19, domestic violence, help-seeking, intimate partner violence, rape, sexual assault

## Abstract

During the first months of the COVID-19 pandemic, governments instituted a series of measures to control the spread of the virus. The measures were widely believed to increase women’s risk of violent victimization, most of which is by an intimate partner. We examined help-seeking during this period in a large U.S. city and used an interrupted time series analysis to assess the effects of three government interventions on domestic violence and sexual assault hotline calls and on “911” calls regarding domestic violence, assault, and rape. Declaration of an emergency appeared to reduce victim calls to the rape crisis hotline and the few “911” calls about rape. School closure was associated with a reduction in “911” calls about assault and rape and victim calls to the domestic violence hotline. Implementation of stay-at-home orders was associated with a gradual increase in domestic violence hotline calls. Although “911” calls regarding assault fell by nearly half, calls to police for domestic violence were unchanged. In sum, there was a decrease in help-seeking for sexual assault and assault in general but not for domestic violence during the initial phases of the COVID-19 outbreak. The analysis underscores the importance of distinguishing between the violence itself, calls to police, and calls to helplines when claims are made about changes over time in violence against women. The opportunities and constraints for each can differ widely under usual circumstances, circumstances that were altered by public health interventions related to the pandemic.

## Background and significance

Social distancing and stay-at-home orders were imposed around the globe in early 2020 in an effort to reduce the spread of the COVID-19 virus. Such measures reduced exposure to people outside the home who may be asymptomatic carriers of the virus and increased exposure to family and other household members. Although these measures were necessary to reduce disease transmission, the combination of fear, isolation, and subsequent economic and social pressures caused experts to fear a “horrifying global surge in domestic violence.” (Guterres, n.d.). Initial reports of this surge, discussed by community organizations worldwide, were followed by examples of how local governments were responding (Guedes et al., 2020) and guidance as to how best to meet victim needs during the pandemic (U.N. Women, n.d.). Assessing the actual, real-time rates of domestic violence during this or any other period is difficult, if not impossible.

Violence against women is endemic, and most of it is at the hands of an intimate partner (Devries et al., 2013). In a population-based national survey of community residents in the United States, 36.4% (41.6 million) women reported that they have, at some point in their lives, experienced contact sexual violence, physical violence (most of it being severe), and/or stalking by an intimate partner (Smith et al., 2018). The survey is conducted annually and, thus, cannot provide a real-time measure of a change in prevalence. Service use, by contrast, can serve as an indicator on a smaller time scale, albeit of a somewhat different phenomena.

Victims of violence can turn to hotlines (also known as helplines) and law enforcement for aid. Hotlines provide information about resources such as emergency shelter, emotional support, and help in problem solving and safety planning. Domestic violence and rape crisis hotline service providers field numerous calls. For example, there were 79,677 calls about domestic violence to one New York City hotline in 2018 (Safe Horizon, n.d.) and 13,931 calls to one rape and battering hotline in Los Angeles in 2019 (Peace Over Violence, n.d.). Even when the number of calls is far lower, as is the case in less populated locales, the service provided is unique: immediate care and support 24 hours a day for victims and survivors and those who want to help them. Law enforcement agencies, in addition to being first-responders to violent incidents, provide on-the-scene intervention and information about protection from abuse orders, emergency shelter, and other resources. Domestic violence is widely believed to be law enforcement’s most common emergency (“911”) call for assistance.

Research on violence against women in the context of disasters, which suggests that domestic violence increases following a disaster and is associated with proximity to its geographic site (Anastario et al., 2009; Campbell et al., 2016; Harville et al., 2011; Lauve-Moon & Ferreira, 2017; Rao, 2020; Schumacher et al., 2010) is of limited usefulness when it comes to COVID-19. For example, during Hurricane Katrina, many people were separated from and frantically searching for family members as well as experienced major property loss and protracted displacement when they left their homes and New Orleans itself. COVID-19 presented substantially different stressors from those experienced in natural disasters; notably, time with family members increased while contact with people outside the home was severely curtailed.

Multiple life stressors are associated with increased risk of assaulting and abusing an intimate partner (e.g., Cano & Vivian, 2001; Roberts et al., 2011; Schwab-Rees et al., 2016), and such stressors were evident in the beginning phases of the pandemic. Job loss and firearm purchases provide two such examples. Record unemployment claims were filed and financial stressors increased in the population as stay-at-home policies and restrictions on in-person gatherings continued for months. These changes increased food insecurity, housing nonpayment, and utility nonpayment, which research indicates are associated with a higher likelihood of perpetrating multiple forms of physical violence against an intimate partner (e.g., Schwab-Reese et al., 2016). In addition, firearm sales spiked during the pandemic; nine of the ten weeks with the most background checks have occurred since COVID-19 began (FBI, n.d.). Firearms are the most common weapon intimate partners use to kill their partner (Fridel & Fox, 2019), and the acquisition of a firearm is associated with women’s higher risk of being murdered in the home (Wintemute et al., 1999). Moreover, abusers use guns in ways that do not result in death, most commonly, to intimidate and coerce their partners (Sorenson, 2017). Thus, some circumstances associated with the pandemic suggest that an increase in violence against women would be likely.

Routine activity theory (Cohen & Felson, 1979) can help conceptually to unpack broad claims about the pandemic’s effects. The theory has been applied for many years to multiple forms of violence against women including domestic violence (Mannon, 1997), rape (Belknap, 1987), and stalking (Mustaine and Tewksbury, 1999).

The theory has three interacting components: a motivated perpetrator, an appropriate target, and an absence of obstacles. Interventions to curb the pandemic could affect all three. For example, stay-at-home orders risk placing a motivated perpetrator more frequently in close proximity to an intimate partner, with children and other members of the household perhaps serving as obstacles or even additional targets. Impacts on household dynamics can differ in terms of the actual violence, calls to first responders, and calls to hotlines. As a result, patterns over time can differ, too. Thus, comprehensive claims about the pandemic’s effects on violence should be met with some skepticism.

Systematic data regarding violence against women during the initial phases of COVID-19 in the United States are just beginning to emerge. News reports, which tended to contradict one another even when issued at roughly the same time, were the primary source of information for several months. A review of studies that covered a time period similar to that of the present investigation found no consistent pattern of findings when it came to domestic violence (Peterman et al., 2020). U.S. studies focused on more than a dozen cities, examined follow-up periods ranging from two to 12 weeks for the one or two interventions studied, used daily or weekly tallies, and examined law enforcement data, either police calls for service or police-recorded crime incidents. The impact upon domestic violence of COVID-19 and interventions designed to reduce its spread is far from a settled question.

We undertook the present investigation to examine violence against women in the context of COVID-19 and how three sequential government interventions—declaration of an emergency, school closure, and stay-at-home orders—may have impacted the help-seeking of victims of violence during the beginning phases of the pandemic in the United States. The article also contributes to the literature in that it examined the number of daily calls to several types of services—domestic violence hotline, sexual assault hotline, and law enforcement—to which assaulted and abused women are likely to turn for help.

## Methods

The daily number of telephone calls to law enforcement and directly relevant social service agencies comprised the data. Access to daily numbers was essential to identify rapid changes, as the initial intervention to curb the pandemic was introduced with little notice, went into effect almost immediately, and was followed very shortly afterward by other interventions. This pattern of intervention occurred across the nation as well as at the site of our investigation, Philadelphia, Pennsylvania.

Philadelphia was an ideal setting for the research because, unlike many other locales, the city and county are contiguous and are served by one domestic violence hotline (collaboratively staffed by four agencies), one sexual assault crisis hotline, and one police department. As such, we could gather information quickly from all providers of the services.

No personal, identifying information was provided to the researchers; in fact, even basic demographic information generally is not available for such calls. For example, “911” operators do not routinely ask for or record a caller’s age, race and ethnicity, gender, or any other demographic characteristic when determining the need and priority assigned for emergency response. Thus, the study was exempted from IRB review.

### Data Sources

The number of calls each day from January 1, 2020, through May 30, 2020, were obtained from the domestic violence hotline and the sexual assault hotline. Few of the agencies routinely offer the option of communicating via text, and very few requests for help were fielded via text messaging. Thus, we focused on telephone calls. The data included the number of calls from victims, friends and relatives of the victim, third-party professionals (e.g., health care providers), and a few other less common callers. Because there were data for 150 days, there were 150 observations each for the domestic violence hotline and the sexual assault hotline.

The number of “911” calls for assistance were obtained from the Philadelphia Police Department for each day from January 1, 2020, through May 15, 2020, for several types of calls. We report herein on calls that the dispatcher identified as domestic violence and, for comparison purposes, assault and rape. It is important to note that law enforcement defines domestic violence broadly to include incidents “characterized by intimacy, familial ties, or a shared household” (Dempsey, 2006). Previous analysis of “911” domestic violence calls in the city indicates that nearly 60% of the dispatch calls are screened out because they are unfounded or determined to be another type of call (Sorenson, 2017). Of the remaining calls, responding officers considered 65.0% to be cases of intimate partner violence (Sorenson, 2017). Thus, about one-fourth of all “911” calls for domestic violence are designated as incidents of intimate partner violence. There were 135 observations for the 135 days of calls to police.

By looking at emergency calls to police and calls to rape crisis and domestic violence hotlines, we were able to capture a fuller range of help-seeking than had we studied only one type of violence or one type of service provider.

### Interventions

We examined the effect of the three government interventions related to COVID-19 that were implemented during the study period: declaration of a disaster emergency (statewide; effective Friday, March 6, 2020), school closures (citywide; effective Monday, March 16, 2020), and a stay-at-home order (citywide; effective Monday, March 23, 2020). These three interventions were used as predictor variables in the statistical analyses.

### Research Design and Statistical Analysis

Because the entire population was subject to the government measures, the impact of the three interventions was assessed using an interrupted time series design. First popularized by Campbell and Stanley more than 50 years ago (1963) and considered a “strong” quasi-experimental design, it addresses abrupt causes that can sharply alter *trends* over time, unlike conventional before-after designs that include data at one point in time before an intervention and one point in time after. Before-after designs cannot properly represent time *trends* and are considered “weak.” By controlling for longitudinal confounders that change gradually, many competing causal explanations can be properly discarded with interrupted time series designs. Such designs also have the capacity to capture the impact of interventions in a dynamic form that allows for somewhat more gradual time trend changes even when the intervention is sudden. Furthermore, several effective diagnostic tools can help in the initial model specification and evaluations of the results.

One of the major and often unrecognized problems with many time series analyses is that the empirical search for an appropriate model specification is a form of model selection, which can invalidate statistical inference (Berk et al., 2013; Leeb & Pötscher, 2006). In our analyses, these difficulties were circumvented by specifying in advance a sensible time series model and not altering it throughout the analyses. This was possible because we knew the timing of the interventions, anticipated particular dynamic changes in the response variable, and used subject-matter insights to specify a seasonal ARIMA formulation for the residuals. The results of the specification were evaluated for each interrupted time series analysis undertaken (Box et al., 2016). In each case, there was no evidence of misspecification of the mean function or of the variance function; the residuals were effectively white noise.

Three transfer step-functions—one for each government intervention—were included. The common model included three components:

1. *An autoregression term with a lag of one day*. The lag was applied because calls to police and hotlines arrive 24 hours a day 7 days a week and events that might impact those calls can spill over from late one night to early the next morning, which introduces serial dependence between calls on adjacent days. The autoregression term can address this dependence and allow for a less abrupt change in response to interventions.
2. *An autoregression term at a lag of 7 days*. This lag was applied to take into account effects of the day of the week. One can expect, for instance, that the number of calls on Saturdays will be more like the number of calls on other Saturdays than, for example, calls on Wednesdays. We used an autoregression formulation to allow a less abrupt transition from winter to spring.
3. *The step functions coded 0 before each intervention and 1 thereafter*. Step functions, when included with one or more autoregression terms, can capture a sharp change in level, a sharp change in slope, or a gradual change in level, depending on the estimated value of the autoregression coefficients, as in 1 and 2 above.

We used the *arimax* procedure in the *TSA* library in R. In each analysis, the fitting algorithm converged quickly with no error messages and diagnostic tests revealed no problems. For response variables with a very low mean number of calls, we used the square root transformation of the response variable to improve inferential properties (Sivan et al., 2016). Consistent with recent recommendations, we adopted a critical *p*-level of .005 rather than .05 (Benjamin et al., 2018). Results are presented primarily in graphical form and are organized by the type of service sought.

## Results

There were 4,587 calls to the domestic violence hotline during the first five months of 2020: 69.1% were from victims, 10.5% from third-party professionals, 9.6% were hang-up calls, 4.7% were from individuals seeking other services, 3.3% were from friends or relatives of victims, and the remaining 2.9% were wrong numbers, prank calls, or from an abuser. During the same time period, there were 1,091 calls to the rape crisis hotline, 89.7% of which were from victims, 5.3% from friends or relatives, and the remaining 5.1% from third-party professionals or persons seeking other services. From January 1, 2020, through May 15, 2020, the police department received 30,724 calls for assistance for domestic violence (over 7,500 of which are expected, as previously noted, to have been determined to be for intimate partner violence), 4,322 for assault, and 583 for rape. The tally of “911” calls did not include information regarding who placed the call. Analysis of the hotline data was limited to calls placed by victims, who comprised the majority of callers.

**Figure 1.**
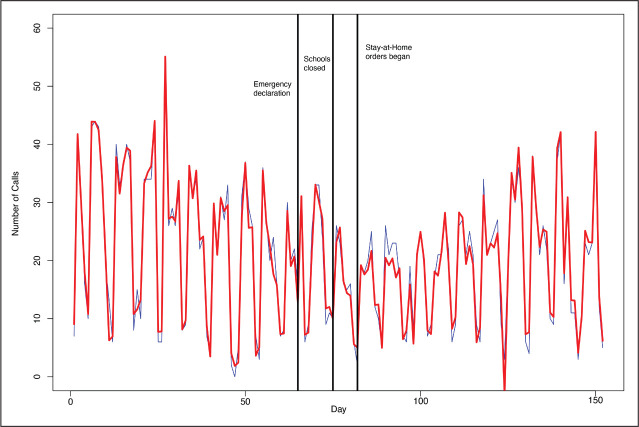
Victim calls to domestic violence hotline, interrupted time series analysis, Philadelphia, January 1, 2020–May 30, 2020.

*Note*. Blue = Actual count; Red = Fitted count.

The mean number of daily calls to the domestic violence hotline was 20.1 (*SD* = 11.7). Relative to the mean, the natural day-to-day variation was substantial, in part, because the number of calls often was notably fewer on weekends and holidays. The day-to-day variation is apparent in Figure 1; it is challenging to determine what is systematic and what is random. Victim calls to the domestic violence hotline dropped by an estimated 6.9 calls each day (*p* = .06) when schools closed and gradually returned to nearly their previous levels after stay-at-home orders began. But the null hypothesis cannot be rejected at the .05 level, let alone the .005 level, indicating that these changes in call volume were not statistically significant.

**Figure 2.**
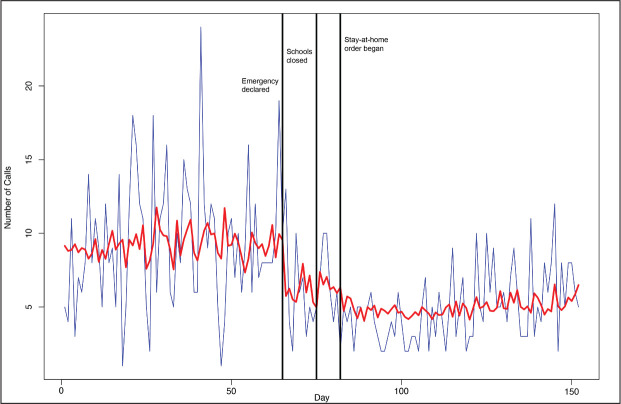
Victim calls to rape crisis hotline, interrupted time series analysis, Philadelphia, January 1, 2020–May 30, 2020.

*Note*. Blue = Actual count; Red = Fitted count.

The mean number of daily calls to the rape crisis hotline was 1.9. Figure 2 displays a discernable reduction of 1.1 calls per day (*p* = .02) when the governor declared a disaster emergency and, despite some variability, the number of calls did not rebound. Given that we adopted a *p*-level of .005, the obtained *p*-level of .02 should be interpreted as suggestive.

**Figure 3.**
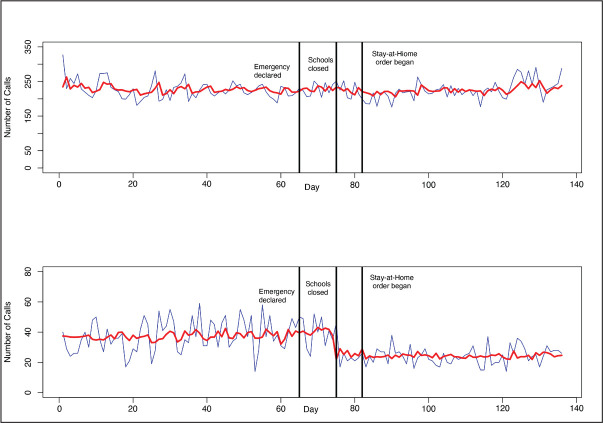
“911” calls for domestic violence (top) and assault (bottom), interrupted time series analysis, Philadelphia, January 1, 2020–May 15, 2020.

*Note*. Blue = Actual count; Red = Fitted count.

The mean number of daily “911” calls for assistance at the scene of a domestic violence incident was 226. The top graph in Figure 3 reveals little systematic change over time in the number of calls, and none of the null hypotheses for the three interventions were rejected at the .05 level or even at the .10 level. The mean number of daily “911” calls for assault was 31.8. In contrast to the pattern for “911” calls for domestic violence, the bottom graph in Figure 3 shows that daily calls for assault, already substantially lower than domestic violence calls, dropped by nearly half (14.4 calls each day; *p* = .005) when the schools closed and remained at that lower level for the duration of the study period.

The mean number of daily “911” calls for rape was 4.3. The pattern of findings was very similar to that for the assault calls, that is, there was a reduction in calls immediately after the emergency declaration. Using only the indicator variable for the emergency declaration, there was a substantial reduction of 2.3 calls each day (*p* = .0005).

Service providers perceived an uptick in the number of hang-up calls since the start of COVID-19, which might indicate the inability to conduct a conversation because, for example, the abuser entered the room. However, the data did not support their supposition. Hang-up calls are not recorded by the rape crisis hotline and there were no systematic trends for hang-up calls to the domestic violence hotline.

## Discussion

The question of whether violence against women increased during the initial months of COVID-19 does not have a simple answer. There is not a way to assess changes in the violence and abuse itself on a daily basis; as such, we examined requests for help that were made to relevant agencies. Not all women seek help for their abuse experiences. National data indicate that only 2.1% of women who had been abused by a partner at some point in their lives reported that they contacted a crisis hotline (Black et al., 2011). In addition, barriers to disclosing intimate partner violence to law enforcement and other service agencies vary by cultural factors and demographic characteristics of the caller (i.e., race, ethnicity, language proficiency, concerns about structural bias; Rizo & Macy, 2011; Tillman et al., 2010) that were not available in the data that were available to us. Nonetheless, service use is an important starting point to understand the implications in real time of three sequential government interventions related to the pandemic. Interpretations of the findings offered by the victim service provider agencies helped contextualize the results.

When schools were closed because of COVID-19, calls to the city-wide domestic violence hotline dropped quickly and returned to their previous level when stay-at-home orders were instituted. We surmise that this pattern does not reflect a temporary decrease in domestic violence but the reality of women’s lives: women continue to bear primary responsibility for children. As such, we surmise that rapid changes in household structure and routine activities (e.g., child care, food preparation, and home-schooling) impacted hotline utilization during the initial adjustment period. An additional conjecture, drawing on routine activities theory, is that partners were able to stay out of the home for work or other reasons, which initially led to a decrease in domestic violence itself when the children could not go to school. After the partner was required to stay at home a few days later, calls resumed at their usual level.

An unexpected finding was the sudden reduction in calls to the city-wide rape crisis hotline when the governor declared an emergency and the subsequent stable lower numbers after stay-at-home orders were implemented. Consistent with routine activities theory, reduced exposure and a change in the population may be relevant. According to a national self-report victimization survey, about half of rapes are perpetrated by individuals who are acquainted with or strangers to the victim (Black et al., 2011), that is, potential perpetrators with whom there would be little exposure while stay-at-home orders were in effect. Victim service providers surmised that the observed pattern of findings also might reflect a change in the population of help-seekers, specifically, college students. Rates of campus sexual assault are high and acquaintances are the most common perpetrator (Cantor et al., 2020). Declaration of a disaster emergency corresponded closely to the start of spring break for the many colleges and universities in the Philadelphia region. Shortly thereafter, students were asked to leave their schools because of COVID-19; most left the region and returned to their parental home, leading many to simply no longer be in the geographic population.

Calls to police for domestic violence remained unchanged during the study period. By contrast, “911” calls for assault began to drop shortly before schools were closed and stayed at a substantially lower level. These findings may indicate that interventions associated with COVID-19 reduced the density of potential perpetrators and potential victims and limited opportunities for at least some forms of street crime (i.e., assault). The same did not apply to households.

There is no evidence that hang-up calls, perhaps an indicator of not being able to safely complete a conversation, increased to the domestic violence hotline. Nor is there evidence that the number of calls were affected by a marked change in phone service. In fact, in mid-March 2020, the Federal Communications Commission asked broadband and telephone service providers to maintain service to those who were unable to pay their bills due to COVID-19 related disruptions; hundreds of companies signed on (Federal Communications Commission, 2020). The action is important given that intimate partner violence is associated with disconnected phone service (Schwab-Reese et al., 2016).

### Study Strengths and Limitations

Two strengths of the current study, as previously mentioned, are that we were able to gather information from all relevant service providers in one large U.S. city and to do so quickly. Moreover, we included data on sexual assault, a form of violence against women that, with rare exception (e.g., Yoshihama et al., 2019) has not been addressed in the scientific literature in the context of disasters or by the news media in the context of COVID-19.

Research using the full population of calls for service, as we did here, is uncommon. Although there is a substantial literature on demographic characteristics that are associated with domestic violence and sexual assault, we, like others who use administrative data about calls, are unable to comment on the characteristics of the individuals who placed the calls. Such information is not core to the function to be fulfilled (e.g., to dispatch a police unit) and, as such, is not routinely gathered.

Some might criticize our work because it does not address child abuse. We agree that calls to child abuse hotlines could reasonably be expected to change during stay-at-home orders. They were not investigated, however, because we focused on the unique experiences of adults and because calls to child abuse hotlines come primarily from third-party reporters, whereas calls to domestic violence hotlines, rape crisis hotlines, and the police come primarily from victims.

We were able to assess change in help-seeking but not a change in the need for such services (i.e., a change in the actual phenomena) which, to be fair, there currently is not a way to assess in real time. The data allowed us to assess a change in help-seeking for the population but not to assess changes, if any, in the nature or severity of the violence that was inflicted. Qualitative studies will be needed to explore whether and how stay-at-home orders, in particular, affected violence and safety. In addition, the law enforcement definition of domestic violence is broad and, as noted previously, includes incidents in addition to intimate partner violence. Our estimate that one-fourth of the calls are later determined to be for intimate partner violence is based on data from a few years ago and might not hold in the context of the pandemic.

## Conclusions

Isolation, a hallmark of violence in relationships, as well as economic strain was heightened when stay-at-home orders began to be issued around the globe. In contrast to reports of a marked increase or decrease in calls to helplines serving abused and assaulted women (Guterres, n.d.; Southall, 2020; World Health Organization, 2020) and increases in calls to police (Boserup et al., 2020), we found little change in calls to a city-wide domestic violence hotline and in domestic violence calls to police. There was, however, a reduction in domestic violence calls for a brief period that might be related to traditional gender roles associated with childcare and possibly reduced exposure to a partner at that brief time. There is evidence that sexual violence, at least as represented by calls to the city-wide rape crisis hotline and police, decreased. How the demand for services change as restrictions lift remains to be seen.

The COVID-19 pandemic likely created special difficulties for services that are routinely available for domestic violence victims. Safe housing is an especially important example. Reluctance to go to an emergency shelter is understandable in the context of what is known about the transmission of COVID-19. Existing policies and practices, such as providing temporary housing vouchers when shelters are full, could be expanded if private or government funding was made available.

Consistent with routine activities theory, interventions associated with COVID-19 may have decreased opportunities for street crime, but not for violence in the home (except possibly very briefly and indirectly measured by hotline calls). Vulnerable populations need to be taken into account when devising public health interventions. Restrictions on movement during the initial phases of the COVID-19 outbreak in the United States largely ignored the needs of abused and assaulted women; stay-at-home orders increased exposure to an abusive partner without providing additional supports for victims. In addition, the findings highlight the importance of examining multiple data sources when addressing a seemingly simple question.

## Author Biographies

**Susan B. Sorenson**, PhD, is professor of Social Policy, professor of Health and societies, and senior fellow in Public Health at the University of Pennsylvania where she also directs the Ortner Center on Violence & Abuse. A public health researcher and clinical psychologist, she has published widely in the epidemiology and prevention of violence.

**Laura Sinko**, PhD, is a postdoctoral fellow at University of Pennsylvania through the National Clinicians Scholars Program. She is a PhD-prepared nurse and currently works as sexual assault nurse examiner. She is a clinical trauma specialist with research interests in trauma-informed care and the trauma recovery process for survivors of gender-based violence.

**Richard A. Berk**, PhD, is a member of the Department of Statistics and the Department of Criminology at the University of Pennsylvania. He is an elected fellow of the American Statistical Association and the American Association for the Advancement of Science. Professor Berk is an applied statistician who often works on criminal justice applications.
